# Population-Based Cohort of Children With Parapneumonic Effusion and Empyema Managed With Low Rates of Pleural Drainage

**DOI:** 10.3389/fped.2021.621943

**Published:** 2021-07-21

**Authors:** Luis Moral, Teresa Toral, Agustín Clavijo, María Caballero, Francisco Canals, María José Forniés, Jorge Moral, Raquel Revert, Raquel Lucas, Ana María Huertas, María Cristina González, Belén García-Avilés, Mónica Belda, Nuria Marco

**Affiliations:** ^1^Pediatric Respiratory and Allergy Unit, Alicante University General Hospital, Alicante Institute for Health and Biomedical Research (ISABIAL), Alicante, Spain; ^2^Department of Pediatrics, Marina Baixa Hospital, Villajoyosa, Spain; ^3^Department of Pediatrics, Vinalopó University Hospital, Elche, Spain; ^4^Department of Pediatrics, Elche University General Hospital, Elche, Spain; ^5^Department of Pediatrics, Virgen de la Salud University General Hospital, Elda, Spain; ^6^Faculty of Medicine, Miguel Hernández University, Sant Joan d'Alacant, Spain; ^7^Department of Pediatrics, Alicante University General Hospital, Alicante, Spain; ^8^Department of Pediatrics, Marina Salud Hospital, Denia, Spain; ^9^Department of Pediatrics, Sant Joan d'Alacant University Clinical Hospital, Sant Joan d'Alacant, Spain; ^10^Department of Pediatrics, Virgen de los Lirios Hospital, Alcoy, Spain; ^11^Department of Pediatrics, Vega Baja Hospital, Orihuela, Spain

**Keywords:** parapneumonic effusion, pleural empyema, pleural drainage, population-based study, length of stay

## Abstract

**Introduction:** The most appropriate treatment for parapneumonic effusion (PPE), including empyema, is controversial. We analyzed the experience of our center and the hospitals in its reference area after adopting a more conservative approach that reduced the use of chest tube pleural drainage (CTPD).

**Methods:** Review of the clinical documentation of all PPE patients in nine hospitals from 2010 to 2018.

**Results:** A total of 318 episodes of PPE were reviewed; 157 had a thickness of <10 mm. The remaining 161 were 10 mm or thicker and were subdivided into three increasing sizes: PE+1, PE+2, and PE+3. There was a strong relationship between the size of the effusion and complicated effusion/empyema, defined by its appearance on imaging studies or by the physical or bacteriological characteristics of the pleural fluid. The size of effusion was also strongly related to the duration of fever and intravenous treatment and was the best independent predictor of the length of hospital stay (LHS) (*p* < 0.001). CTPD was placed in 2.9% of PE+1 patients, 19.3% of PE+2, and 63.9% of PE+3 (*p* < 0.001). The referral of patients with PE+1 decreased over time (*p* = 0.033), as did the use of CTPD in the combined PE+1/PE+2 group (*p* = 0.018), without affecting LHS (*p* = 0.814). There were no changes in the use of CTPD in the PE+3 group (*p* = 0.721).

**Conclusions:** The size of the PPE is strongly correlated with its severity and with LHS. Most patients can be treated with antibiotics alone.

## Introduction

Parapneumonic effusion (PPE), including simple and complicated effusion and empyema, is the most common complication of pneumonia in children. Most clinical studies on PPE are reference hospital-based, and there is currently no consensus on the most appropriate treatment (1–3). Small PPE (<10 mm thick) can usually be managed conservatively. Several clinical trials have been carried out to verify the most appropriate method to drain complicated effusion and empyema (CE/E) (4, 5). Conservative treatment of CE/E is based primarily on antibiotics, restricting chest tube pleural drainage (CTPD) or video-assisted thoracoscopy to the most severe or treatment-resistant cases. This approach has never been addressed in clinical trials, although some centers have published their experience with good results (6, 7). Little is known about risk factors for prolonged length of hospital stay (LHS) (8).

In 2010, we changed our approach to treating CE/E. The decision to use CTPD was personalized according to practicing physicians' (non-standardized) clinical criteria (e.g., persistent septic appearance, marked respiratory distress) rather than the radiological criteria used previously (size and complexity of effusion). As a result, draining of CE/E cases dropped from 83% in the period 2005–2009 to 47% in 2010–2013, without significant differences in outcomes, including LHS (9). After 9 years we wanted to review the treatments and outcomes of our patients, but more than 70% of them came from the eight community hospitals (CH) to which we serve as a reference. Those eight CH are similar in size and all have a dedicated pediatric service, but they lack pediatric interventional radiology services, pediatric surgery and pediatric intensive care, so many patients with PPE must be transferred to our center. As a consequence of our shift to a conservative treatment, those CH might have also progressively changed their referral criteria, raising the severity threshold and reducing the number of transfers. There were no hospital guidelines for antibiotic selection or transfer criteria, which were at the discretion of practicing pediatricians. The size of the PPE was usually perceived as one important objective factor for transfer and management decisions. To avoid the biased view of a reference center, we decided to review all the patients attended in the nine hospitals of our geographic area, including those not transferred. The main objectives of this study were (a) to describe the characteristics, treatment and outcomes of a population-based cohort of children admitted with PPE after adoption of the conservative treatment policy, and (b) to identify factors associated with the LHS in patients with large PPE (≥10 mm thick).

## Patients and Methods

### Selection of Patients

Patients were recruited from our hospital and the other eight public CH, together covering a population of just over 250,000 children under the age of 15. Ours is the only reference center for all children in the area requiring invasive procedures or intensive care. Episodes of PPE that met the following three criteria were reviewed: (a) patients under 15 years of age at the time of admission; (b) hospitalized from 1 January 2010 to 31 December 2018; and (c) diagnosed with pleural effusion or empyema, either as the primary or secondary diagnosis (ICD9 diagnostic codes 510.0, 510.9, 511.1, 511.81, 511.89, 511.9 or ICD10 diagnostic codes J86.0, J86.9, J90, J91, J91.8). Medical records were individually reviewed to rule out patients who met any of the exclusion criteria: (a) non-infectious pleural effusion; (b) tuberculosis; (c) nosocomial pneumonia; (d) concurrent severe diseases that influenced the treatment, clinical course and LHS more than the PPE itself; (e) patients transferred to distant hospitals outside the study area. Patients who met the eligibility criteria were selected to complete the case report form by reviewing their medical history and image studies.

### Variables

We recorded patient data at admission, including sex, age (years old), year and month of admission, previous diseases, days of fever and antibiotics administered before hospitalization. Vaccination status was not available for most of the patients and was not analyzed. The worst recorded values of blood leukocytes, neutrophils, C-reactive protein, sodium and urea during hospitalization were reviewed. For an easy and simple multicenter retrospective classification, the size of the PPE was divided into four groups, based on the maximum thickness of the effusion observed in any diagnostic imaging: <10 mm (PE–), 10–20 mm (PE+1), >20 mm but not massive (PE+2), and massive (PE+3), the latter considered as the complete or almost complete opacification of the affected hemithorax. To perform some of the analyses, all patients with effusions of 10 mm or greater (PE+1, PE+2, and PE+3) were pooled into a single group (PE+). The total number of leukocytes, percentage of neutrophils, and protein and glucose concentration in the pleural fluid (PF) were recorded when available. Blood culture and PF culture results were recorded. The etiologic agent was considered confirmed when the growth of a characteristically pathogenic bacterium was reported in the blood or PF culture, after exclusion of presumed contaminant or doubtfully pathogenic bacteria (10). Other microbiological studies (*Mycoplasma pneumoniae* serology, bacterial antigen detection, viral detection) were not analyzed due to the great variability between participant centers or uncertain interpretation. CE/E was defined by the observation, if available, of echogenic (by ultrasound) or radiopaque (by computed tomography) images inside the pleural effusion, by direct observation of an opalescent or purulent PF, or by growth of any confirmed pathogenic bacteria in the PF, in cases in which a sample was obtained by means of puncture or drainage. Intravenous and oral antibiotics administered and the duration of treatment since admission were retrieved. CTPD placement, administration of oxygen, admission to the intensive care unit and assistance with mechanical ventilation were noted. The presence of pneumothorax and the timing of its detection were recorded as well as the duration of fever during hospitalization. LHS was counted in days from the first admission to a hospital for the episode of PPE until the final discharge, even if it was in a different hospital after having been transferred. Calculation of LHS also included the days spent at home after discharge when readmission was required for the same episode. Data were reviewed and recorded by investigators from each of the participating hospitals. In case of any doubt or incongruity, especially about diagnostic imaging, the main investigator reviewed the conflicting information to make a decision and ensure consistent criteria.

### Statistical Analysis

The data collected in the case report forms were entered into a database for statistical processing using the SPSS v.26 and the R v.4.0.2. programs. Statistical significance was set at *P* < 0.05.

A descriptive analysis of the whole cohort was performed, calculating the frequencies and percentages for the qualitative variables, and the median and the interquartile range for quantitative variables. Pearson's χ^2^ test (or Fisher's exact test in 2×2 tables) was used for the comparative analysis of qualitative variables, and the Mann-Whitney *U*-test or the Kruskall-Wallis *H*-test for the quantitative variables, as appropriate. The χ^2^ test for linear trend was used for qualitative variables with ordered values (size of the effusion, year of hospitalization).

In patients with PE+, we analyzed mean LHS according to each explanatory categorical variable, using Welch's robust test or the Mann-Whitney *U*-test, as appropriate. For explanatory quantitative variables, we calculated Spearman's correlation coefficient. Finally, we fitted a log-linked Gamma generalized linear model to explain the LHS. This variable is not symmetrical nor is it normally distributed, and a linear model does not meet the goodness-of-fit criteria. The Gamma model does not require parametric distributions, and it is a good alternative for performing transformations of the response variable (11). The log-linked function means that the coefficients calculated are exponential, so the interpretation is similar to that of an odds ratio estimated by logistic regression: if it is over 1, the explanatory variable is associated with an increase in the mean LHS, whereas values of <1 mean that the variable is associated with a decrease. The effect magnitude is calculated as [1 – exp(beta)] × 100%, which is the percentage of mean change in LHS associated with the corresponding variable. A complete table with these coefficients is shown for the final model, fitted using a stepwise selection approach from the baseline variables, according to the Akaike Information Criterion (AIC). We present the results of the deviance test for goodness-of-fit as well as residual plots to test the linearity of the predictors and the appropriateness of the Gamma model.

### Ethics

The study was approved by the research ethics committee of our hospital.

## Results

We evaluated 337 cases of non-tuberculous PPE; 14 were excluded from analysis due to nosocomial pneumonia or concurrent severe diseases that influenced the treatment, clinical course, and LHS more than the PPE itself (Supplementary Table 1). Another five patients were transferred to distant hospitals for unrelated reasons and were also excluded. Thus, a total of 318 PPE episodes were included: 157 patients (49.4%) had a PE- and 161 (50.6%) a PE+, distributed as 68 (21.4%) PE+1, 57 (17.9 %) PE+2 and 36 (11.3%) PE+3. Table 1 presents the descriptive analysis of the cohort. Seventy-six patients (23.9%) had previous or concomitant diseases (Supplementary Table 2). The strong relationship between the size of the effusion and CE/E is reflected in Table 1. A significant correlation was also observed between the size of the effusion and the results of blood (but not PF) tests. The total number of leukocytes in PF varied widely and was not related to the size of the effusion (*p* = 0.621). The result of blood or PF culture was documented in 259 patients (81.4%). The etiologic agent was considered confirmed in 24 patients (7.4% of the total and 9.1% of those with known cultures): *Streptcoccus pneumoniae* in 18, *Streptococcus pyogenes* in 5, and *Haemophilus influenzae* in 1 patient. In 19 patients, bacteria that grew in the blood or PF culture were presumed contaminant or doubtfully pathogenic (Supplementary Table 3). The duration of treatment and the antibiotics used, the administration of oxygen, and admission to intensive care, were related to the size of the effusion, as observed in Table 1. Pneumothorax was detected in 15 patients, all of them with PE+2 (5.3% of this group) or PE+3 (33.3% of this group), but only four had pneumothorax before CTPD placement. Both the duration of fever and LHS were strongly correlated with the size of the effusion (Table 1).

**Table 1:** Patient characteristics, treatment, and outcomes separated by size of effusion.

	**Total**	**Size of effusion**						
		**All patients**			**PE+ patients**			
		**PE–**	**PE+**	**p^a^**	**PE+1**	**PE+2**	**PE+3**	**p^b^**
	***n* = 318**	***n* = 157 (49.4 %)**	***n* = 161 (50.6 %)**		***n* = 68 (21.4 %)**	***n* = 57 (17.9 %)**	***n* = 36 (11.3 %)**	
**Patient data at admission**								
Male sex	176 (55.3 %)	89 (56.7 %)	87 (54.0 %)	0.653	32 (47.1 %)	35 (61.4 %)	20 (55.6 %)	0.284
Age (years)	4 (2–7)	5 (2–7)	3 (2–7)	0.103	3 (2–5)	4 (2–8)	3 (2–6)	0.270
History of asthma	45 (14.2 %)	29 (18.5 %)	16 (9.9 %)	0.036^*^	11 (16.2 %)	2 (3.5 %)	3 (8.3 %)	0.104
History of neurological diseases	17 (5.3 %)	8 (5.1 %)	9 (5.6 %)	1	4 (5.9 %)	1 (1.8 %)	4 (11.1 %)	0.432
Fever before admission (days; *n* = 307)	4 (2–6)	4 (2–7)	4 (2–6)	0.419	3 (2–5)	4 (2–6)	4 (3–6)	0.348
Antibiotics before admission (*n* = 314)	135 (43.0 %)	60 (38.5 %)	75 (47.5 %)	0.112	35 (52.2 %)	25 (43.9 %)	15 (44.1 %)	0.372
Antibiotics before admission (days; *n* =305)	0 (0–3)	0 (0–2)	0 (0–3)	0.114	1 (0–3)	0 (0–3)	0 (0–3)	0.747
Predominantly left effusion	162 (50.9 %)	79 (50.3 %)	83 (51.6 %)	0.211	36 (52.9 %)	27 (47.4 %)	20 (55.6 %)	0.711
**Complicated effusion/empyema**				<0.001^*^				<0.001^*^
Unknown	107 (33.6 %)	89 (56.7 %)	18 (11.2 %)		14 (20.6 %)	3 (5.3 %)	1 (2.8 %)	
No	89 (28.0 %)	59 (37.6 %)	30 (18.6 %)		23 (33.8 %)	6 (10.5 %)	1 (2.8 %)	
Yes	122 (38.4 %)	9 (5.7 %)	113 (70.2 %)		31 (45.6 %)	48 (84.2 %)	34 (94.4 %)	
**Blood results**								
Leukocytes (10^9^/L; *n* = 311)	17.8 (11.8–24.9)	15.2 (10.2–22.4)	19.7 (13.7–27.8)	<0.001^*^	17.2 (12.0–23.6)	20.0 (15.2–28.8)	26.7 (17.0–31.2)	0.002^*^
Neutrophils (10^9^/L; *n* = 307)	12.8 (7.7–19.3)	11.0 (6.0–16.9)	15.3 (9.5–21.0)	<0.001^*^	12.1 (8.4–18.7)	15.3 (9.9–22.0)	18.9 (12.6–26.2)	0.007^*^
C-reactive protein (mg/dL; *n* = 311)	17.8 (6.4–29.3)	10.1 (4.6–20.4)	24.1 (13.6–33.6)	<0.001^*^	21.8 (8.5–32.8)	21.3 (15.3–32.9)	27.7 (22.1–35.4)	0.056
Sodium (mmol/L; *n* = 283)	135 (133–137)	137 (133–138)	134 (132–136)	<0.001^*^	135 (133–137)	134 (131–136)	133 (131–135)	0.036^*^
Urea (mg/dL; *n* = 273)	23 (18–30)	21 (17–27)	24 (19–33)	0.010^*^	22 (17–27)	24 (20–35)	30 (19–40)	0.020^*^
**Pleural fluid results**								
Protein (g/dL; *n* = 36)			4.4 (3.9–4.9)		4.4 (4.4–4.4)	4.4 (3.8–5.1)	4.3 (3.9–4.6)	0.975
Glucose (g/dL; *n* = 36)			2 (1–7)		2 (2–2)	3 (1–5)	1 (1–8)	0.419
Neutrophils (% of leukocytes; *n* = 31)			87 (66–95)		30 (30–30)	81 (68–94)	93 (67–97)	0.243
**Confirmed positive culture** (*n* = 259)^†^	24 (9.3 %)	5 (4.5 %)	19 (12.8 %)	0.029^*^	6 (9.8 %)	4 (7.8 %)	9 (25.0 %)	0.055
**Intravenous treatment**								
Amoxicillin/ampicillin	76 (23.9 %)	40 (25.5 %)	36 (22.4 %)	0.599	17 (25.0 %)	12 (21.1 %)	7 (19.4 %)	0.491
Amoxicillin-clavulanic	50 (15.7 %)	24 (15.3 %)	26 (16.1 %)	0.878	9 (13.2 %)	11 (19.3 %)	6 (16.7 %)	0.552
Cefotaxime/ceftriaxone	245 (77.0 %)	101 (64.3 %)	144 (89.4 %)	<0.001^*^	61 (89.7 %)	50 (87.7 %)	33 (91.7 %)	0.838
Vancomycin	66 (20.8 %)	9 (5.7 %)	57 (35.4 %)	<0.001^*^	19 (27.9 %)	28 (49.1 %)	10 (27.8 %)	0.623
Clindamycin	31 (9.7 %)	5 (3.2 %)	26 (16.1 %)	<0.001^*^	12 (17.6 %)	9 (15.8 %)	5 (13.9 %)	0.615
Length of IV treatment (days; *n* = 307)	7 (4–12)	4 (3–6)	11 (8–14)	<0.001^*^	9 (6–11)	12 (9–14)	14 (12–16)	<0.001^*^
**Total length of antibiotic treatment since admission** (days; *n* = 296)	13 (10–17)	11 (9–13)	16 (13–21)	<0.001^*^	15 (12–18)	16 (14–21)	20.5 (17–24)	<0.001^*^
**Other treatments**								
Chest tube pleural drainage	36 (11.3 %)	0 (0.0 %)	36 (22.4 %)	<0.001^*^	2 (2.9 %)	11 (19.3 %)	23 (63.9 %)	<0.001^*^
Oxygen (*n* = 302)	114 (37.7 %)	34 (23.3 %)	80 (51.3 %)	<0.001^*^	20 (31.3 %)	31 (54.4 %)	29 (82.9 %)	<0.001^*^
Intensive care	17 (5.3 %)	3 (1.9 %)	14 (8.7 %)	0.011^*^	0 (0.0 %)	5 (8.8 %)	9 (25.0 %)	<0.001^*^
Mechanical ventilation	9 (2.8 %)	3 (1.9 %)	6 (3.7 %)	0.502	0 (0.0 %)	2 (3.5 %)	4 (11.1 %)	0.006^*^
**Outcomes**								
Length of fever (days; *n* = 302)	3 (1–7)	2 (1–3)	6 (3–10)	<0.001^*^	4 (2–7)	7 (5–11)	10 (6–14)	<0.001^*^
Length of hospital stay (days)	7.5 (5–12)	5 (3–7)	11 (8–15)	<0.001^*^	9 (6–13)	12 (9–15)	15 (11–22)	<0.001^*^

*Number of cases followed by the percentage in parentheses for the qualitative variables, and the median followed by the interquartile range in parentheses for the quantitative variables. The number of cases (n) is specified in the variables for which data were not available for all patients*.

† *Percentages were calculated out of the total of those with a known blood or pleural fluid culture result in each group*.

a *p-value for Fisher's exact test (qualitative variables) or Mann-Whitney U-test (quantitative variables)*.

b *p-value for χ^2^ test for linear trend (qualitative variables) or the Kruskall-Wallis H test (quantitative variables)*.

* *p < 0.05*.

*For definitions of parapneumonic effusion size (PE–, PE+, PE+1, PE+2 and P+3), see text*.

A CTPD was inserted in 36 patients (11.3%), all of them with PE+ (22.4% of this group) or CE/E (29.5% of this group), generally with urokinase (72.2%, no difference in LHS: *p* = 0.614). Only 1 of the 318 patients, who was previously managed with a CTPD, required video-assisted thoracoscopic surgery after readmission for a reinfection of PPE and had the longest LHS, 54 days, including 24 days at home between admissions. Supplementary Table 4 shows a significant decreasing trend in the use of CTPD over time only in the combined PE+1 and PE+2 group. Of the 249 patients initially admitted to the eight CH in our reference area, 80 (32.1%) were transferred to our center: 7/126 (5.6%) PE–, 18/56 (32.1 %) PE+1, 31/43 (72.1%) PE+2, and 24/24 (100%) PE+3. There was a significant decrease in the transfer only for those in the PE+1 group (Supplementary Table 4). There was no variation over time in the duration of intravenous treatment, fever, or LHS in the whole group of children with PPE or specifically in subgroups of PE+ patients (Supplementary Table 5).

Supplementary Tables 6, 7 present the analysis of factors associated with a longer hospital stay in patients with PE+. Table 2 shows the estimation of the multivariable Gamma model, showing that the magnitude of the PPE is the best predictor of LHS, with a pronounced gradient and positive correlation between the two. We also observed a significant relationship between LHS and several explanatory variables, including duration of pre-hospital antibiotic treatment (the longer the treatment, the shorter the hospital stay), C-reactive protein value, and season (trimester). Sex, year of admission, and hospital of origin were not associated with LHS. The model showed a good fit with the data, showing linearity with the predictors and normality of the residuals (Supplementary Data Sheet 1).

**Table 2:** Multivariate Gamma model for the length of hospital stay for patients with PE+.

		**Beta**	**Exp(Betas)**	**CI 95%**	***p*-value**
Intercept		2.138	8.481	(6.32–11.37)	0.000
Size of effusion	PE+1	0	1		
	PE+2	0.298	1.347	(1.152–1.576)	<0.001^*^
	PE+3	0.494	1.639	(1.351–1.989)	<0.001^*^
Trimester of the year	T3	0	1		
	T4	0.233	1.262	(0.976–1.634)	0.076
	T1	0.249	1.284	(1.007–1.636)	0.046^*^
	T2	0.435	1.545	(1.182–2.019)	0.002^*^
Sex	Female	0	1		
	Male	−0.048	0.953	(0.829–1.096)	0.501
Year of admission	2010–2012	0	1		
	2013–2015	0.086	1.090	(0.916–1.297)	0.332
	2016–2018	0.070	1.073	(0.895–1.286)	0.447
First admission hospital	RH	0	1		
	CH1	0.158	1.171	(0.933–1.470)	0.175
	CH2	0.078	1.081	(0.851–1.372)	0.525
	CH3	−0.142	0.868	(0.670–1.124)	0.285
	CH4	−0.031	0.969	(0.760–1.237)	0.801
	CH5–8	0.150	1.162	(0.955–1.413)	0.136
Confirmed positive culture	Yes	0.194	1.214	(0.971–1.519)	0.091
Age	(years)	−0.019	0.981	(0.961–1.002)	0.080
Length of antibiotics before admission	(days)	−0.050	0.952	(0.924–0.980)	0.001^*^
C-reactive protein	(mg/dL)	0.007	1.007	(1.001–1.012)	0.024^*^

*n = 152; Deviance = 23.7 (p < 0.001)*;

* *p < 0.05*.

*T3: July–September; T4: October–December; T1: January–March; T2: April–June. RH, reference hospital; CH, community hospital. For definitions of parapneumonic effusion size (PE+, PE+1, PE+2 and P+3), see text*.

## Discussion

Our study is unique in showing the detailed characteristics of a complete cohort of patients hospitalized for PPE (including empyema) over a wide geographic area and period of time, providing a panoramic view of the full spectrum of the disease without the bias of the perspective of a reference hospital. We have found no similar population-based reports of children with PPE. Furthermore, given the conservative approach to CTPD, this study offers an overview of the evolution of PPE, managed in many cases only with antibiotics.

The size of the PPE is strongly correlated with CE/E, patient characteristics, treatments, and outcomes. Patients with small effusions (PE–) usually have simple effusions, with moderate analytical changes. They rarely require referral or CTPD, and the fever subsides in a few days, which generally limits the length of intravenous treatment and LHS to <1 week. As the size of the PPE increases, other variables related to its severity and to the intensity of treatment simultaneously increase, such as leukocytosis with neutrophilia, C-reactive protein, sodium (decrease) and urea values in the blood, growth of evident pathogenic organisms in the cultures, CTPD placement, need for oxygen therapy, admission to intensive care, and the length of fever, antibiotic treatment and LHS. The analytical values in the PF show few differences related to the size and severity of the effusion, although these data may suffer from selection bias due to the limited number of patients in which a sample was obtained, mostly those undergoing CTPD. The need for mechanical ventilation was similarly rare in large and small effusions. In patients with PE+, the size of PPE is the best independent predictor of the LHS, followed by the maximum level of C-reactive protein. On the other hand, LHS in PE+ patients decreases with increasing days of antibiotic treatment before admission and in those admitted in the summer. To the best of our knowledge, these findings have never been described before.

Before 2010, more than 80% of patients with CE/E underwent CTPD in our center; this percentage markedly decreased after changing the initial approach to treatment, without significant changes in the outcomes, especially in the LHS (9). As a result, most CE/E are being treated only with antibiotics. Treatment of patients with PE+3 changed the least in this period, and most of them are still treated with CTPD, although about one in three are treated conservatively. Clinical guidelines usually recommend the treatment of CE/E using drainage techniques, mainly CTPD or video thoracoscopy (12–14). However, in real life, many differences in daily clinical practice can be observed (1, 2). Many centers adopt a conservative treatment approach, using only antibiotics, at least initially. Epaud et al. reduced the use of CTPD from 52 to 25% by changing to a more conservative approach, with no change in the outcomes (6). Carter et al. reported extensive experience of conservative treatment of empyema, and 52% of their patients, including 23% with mediastinal deviation, were treated with antibiotics alone (7). Picard et al. followed a conservative approach, using only antibiotics in a third of their patients with empyema (15). Proesmans et al. treated 37% of children with empyema with antibiotics alone, and only 8% required further interventions (16). Long et al. treated 27% of children with empyema with antibiotics, and only 3% required a subsequent intervention (17). In the USA, more than half of children with PPE were treated with antibiotics alone, with an upward trend in the last decade, and similar outcomes were achieved across the most interventional and the most conservative centers (18, 19). In other recent studies, no differences were found in children treated conservatively or with drainage procedures (20, 21).

Our study has limitations, mainly related to its observational and retrospective nature, as the data depend on the quality of the records. For variables with missing data (fewer than 318 observations), the dataset may be less reliable than those with data from all patients in the cohort. It is also not possible to record, in a case report form, all the complexity of the clinical course in some patients, which is simplified into the most objective and quantifiable data. The classification of the size of the effusion may be influenced by various factors, such as the technique used (x-ray, ultrasound, computed tomography), patient position, the timing of the studies, or the early or late placement of the CTPD, among others. Moreover, pleural thickness on diagnostic imaging may be imperfectly related to the volume of the effusion (due to loculated effusion, patient age, or other factors). Microbiological studies other than cultures were not systematically performed, and only clearly pathogenic bacteria growing in blood or pleural fluid were considered, resulting in a low rate of etiological diagnosis. However, the multicenter, population-based nature of the study, collecting extensive clinical and radiological data in patients from a large cohort spanning almost a decade, makes the results highly robust.

In patients without significant comorbidities, PPE mortality, including empyema, is practically nil (4, 18, 20–23), and the medium-term prognosis is good (24, 25). Therefore, the objective of treatment is to shorten hospitalization and simplify treatment, thereby reducing iatrogenesis and costs. Observational studies are not useful to know whether there is a difference between conservative and interventional treatment. It would be interesting to know the predictors of a prolonged or complicated course of PPE to identify, at least theoretically, those who could benefit the most from interventional treatment. Studies carried out in this regard have been scarce and inconclusive (8, 15, 23, 26, 27). To conclude, we have verified that the size of PPE is strongly correlated with its severity and with LHS, so it could be used as a prognostic factor and for treatment decision-making. Most patients can be treated with antibiotics alone, even in CE/E. Clinical trials should determine if and which patients may benefit from early treatment with drainage techniques, to significantly reduce the LHS.

## Data Availability Statement

The raw data supporting the conclusions of this article will be made available by the authors, without undue reservation.

## Ethics Statement

The studies involving human participants were reviewed and approved by Comité Ético de Investigación del Hospital General Universitario de Alicante. Written informed consent for participation was not provided by the participants' legal guardians/next of kin because: Retrospective observational research. Authorized by the ethics committee.

## Author Contributions

LM had primary responsibility for protocol development, preliminary data analysis, and writing the manuscript. TT, AC, MC, FC, MF, JM, RR, RL, AH, MG, BG-A, MB, and NM participated in the collection of patients' data and contributed to the writing of the manuscript. All authors contributed to the article and approved the submitted version.

## Conflict of Interest

The authors declare that the research was conducted in the absence of any commercial or financial relationships that could be construed as a potential conflict of interest.

## Abbreviations

PPE: parapneumonic effusion, including empyema
CE/E: complicated effusion and empyema
CH: community hospital
CTPD: chest tube pleural drainage
LHS: length of hospital stay
PE–: parapneumonic effusion <10 mm
PE+: parapneumonic effusion of 10 mm or greater
PE+1: parapneumonic effusion of 10–20 mm
PE+2: parapneumonic effusion >20 mm but not massive
PE+3: massive parapneumonic effusion (complete or almost complete opacification of the affected hemithorax)
PF: pleural fluid.
