# Supplementary material for: Population-Based Cohort of Children With Parapneumonic Effusion and Empyema Managed With Low Rates of Pleural Drainage
Source: Front Pediatr. 2021 Jul 21;9:621943. doi: 10.3389/fped.2021.621943 (PMC8335639; doi:10.3389/fped.2021.621943)
Supplement: Supplementary file 1 [file Table_1.DOCX]

**Table S1.** Patients excluded from analysis due to nosocomial pneumonia or concurrent severe diseases that influenced the treatment, clinical course and length of hospital stay more than the parapneumonic pleural effusion itself.

| **Age (years)** | **Sex (M/F)** | **Main disease** | **Blood culture** | **Size of pleural effusion** | **CTPD** | **PICU** | **Mechanical ventilation** |
| --- | --- | --- | --- | --- | --- | --- | --- |
| **5 patients with nosocomial pneumonia during hospital admission for other reasons** | | | | | | | |
| 1 | F | Myeloid leukemia | *P. aeruginosa* | PE+2 | Yes | No | No |
| 11 | M | Peritonitis secondary to gangrenous appendicitis |  | PE+2 | Yes | No | No |
| 13 | M | Sepsis and iliopsoas myositis | *S. aureus* | PE+1 | No | No | No |
| 14 | M | Severe traumatic spinal cord injury | *E. cloacae* | PE+2 | Yes | Yes | Yes |
| 7 | M | Plasmacytoid dendritic blast cell neoplasia onset | Presumed contaminant | PE- | No | No | No |
| **9 patients with community-acquired pneumonia accompanying severe diseases** | | | | | | | |
| 7 | M | Acute glomerulonephritis |  | PE- | No | No | No |
| 1 | F | Streptococcal septic shock | *S. pyogenes** | PE+1 | Yes | Yes | Yes |
| 2 | F | Hemolytic uremic syndrome | *S. pneumoniae* | PE+1 | No | Yes | No |
| 1 | M | Anaplastic giant cell lymphoma onset |  | PE- | No | No | No |
| 2 | F | Toxic epidermal necrolysis |  | PE- | Yes | Yes | Yes |
| 2 | M | Acute glomerulonephritis |  | PE+2 | No | No | No |
| 5 | M | Streptococcal septic shock | *S. pyogenes* | PE- | No | Yes | Yes |
| 0 | M | Severe combined immunodeficiency onset | *S. epidermidis* | PE- | Yes | Yes | Yes |
| 4 | M | Severe congenital epileptic encephalopathy | *S. hominis-hominis* | PE+1 | Yes | Yes | Yes |

* Blood and pleural fluid culture

CTPD: chest tube pleural drainage

PICU: pediatric intensive care unit

For definitions of the size of pleural effusion (PE-, PE+1, PE+2 and P+3), see text.
