# Supplementary material for: Population-Based Cohort of Children With Parapneumonic Effusion and Empyema Managed With Low Rates of Pleural Drainage
Source: Front Pediatr. 2021 Jul 21;9:621943. doi: 10.3389/fped.2021.621943 (PMC8335639; doi:10.3389/fped.2021.621943)
Supplement: Supplementary file 2 [file Table_2.DOCX]

**Table S2**. Main previous or concomitant diseases of patients admitted for parapneumonic pleural effusion (PPE). Groups are not exclusive and a patient can belong to more than one group.

| **Disease** | **n (%)** | **Comments** |
| --- | --- | --- |
| Asthma | 45 (14.2%) | Including recurrent wheezing |
| Neurological diseases | 17 (5.3%) | Includes patients with moderate or severe psychomotor retardation. Including five patients with Down syndrome |
| Prematurity | 7 (2.2%) |  |
| Heart diseases | 7 (2.2%) | Generally mild and stable congenital heart defects. Including four of the patients with Down syndrome |
| Chickenpox | 4 (1.3%) | Concomitant with PPE |
| Bronchial foreign body | 3 (0.9%) | Detected and removed shortly after the episode in all three cases |
| Previous PPE | 2 (0.6%) | Patient 1: Two episodes (PE- and PE+1) separated by almost 3 years (4 and 7 years old), did not require drainage. They occurred within the study period and were analyzed as two independent episodes.  Patient 2: 9-year-old patient with PE-. Hospitalized at one year of age (before 2010) for PPE that required pleural drainage on that occasion. |

For definitions of PPE size (PE-, PE+1, PE+2 and P+3), see text.
