# Supplementary material for: Population-Based Cohort of Children With Parapneumonic Effusion and Empyema Managed With Low Rates of Pleural Drainage
Source: Front Pediatr. 2021 Jul 21;9:621943. doi: 10.3389/fped.2021.621943 (PMC8335639; doi:10.3389/fped.2021.621943)
Supplement: Supplementary file 3 [file Table_3.DOCX]

**Table S3**. Presumed contaminant or doubtfully pathogenic bacteria growing from blood or pleural fluid cultures in 19 patients.

**Blood culture**

*Micrococcus luteus*

*Moraxella catarrhalis*

*Staphylococcus capitis*

*Staphylococcus epidermidis* (3 patients)

*Staphylococcus hominis** (8 patients)

*Streptococcus mitis* & *Streptococcus oralis*

*Streptococcus parasanguinis*

**Pleural fluid culture**

*Enterococcus faecium*

*Staphylococcus epidermidis*

*Staphylococcus hominis*

*Streptococcus milleri**

* One patient had blood and pleural fluid growth with 2 different bacteria
