# Supplementary material for: Population-Based Cohort of Children With Parapneumonic Effusion and Empyema Managed With Low Rates of Pleural Drainage
Source: Front Pediatr. 2021 Jul 21;9:621943. doi: 10.3389/fped.2021.621943 (PMC8335639; doi:10.3389/fped.2021.621943)
Supplement: Supplementary file 4 [file Table_4.DOCX]

**Table S4**. Triennial trend in the percentage of patients with parapneumonic effusion (PPE) who underwent chest tube pleural drainage (CTPD) and in the percentage of patients who were transferred from the eight community hospitals to the reference hospital.

| YEARs | 2010-2012 | 2013-2015 | 2016-2018 | p-value |
| --- | --- | --- | --- | --- |
| Percentage of patients who underwent CTPD | | | | |
| Total PE+ | 16/59 (27.1) | 9/49 (18.4) | 11/53 (20.8) | 0.408 |
| - PE+1 | 2/28 (7.1) | 0/17 (0) | 0/23 (0) | 0.126 |
| - PE+2 | 8/22 (36.4) | 1/20 (5.0) | 2/15 (13.3) | 0.052 |
| - PE+1 & PE+2 | 10/50 (20) | 1/37 (2.7) | 2/38 (5.3) | 0.018* |
| - PE+3 | 6/9 (66.7) | 8/12 (66.7) | 9/15 (60.0) | 0.721 |
| Percentage of patients transferred from eight community hospitals to the reference hospital | | | | |
| Total PPE | 32/94 (34.0) | 27/77 (35.1) | 21/78 (26.9) | 0.338 |
| - PE- | 3/45 (6.7) | 3/40 (7.5) | 1/41 (2.4) | 0.404 |
| - PE+ | 29/49 (59.2) | 24/37 (64.9) | 20/37 (54.1) | 0.679 |
| - - PE+1 | 10/25 (40.0) | 7/14 (50.0) | 1/17 (5.9) | 0.033* |
| - - PE+2 | 13/18 (72.2) | 9/15 (60.0) | 9/10 (90.0) | 0.449 |
| - - PE+3 | 6/6 (100) | 8/8 (100) | 10/10 (100) | 1 |

The cells express the quotient of patients who underwent CTPD or who were transferred in each 3-year period and each group (percentage indicated in brackets).

* p < 0.05

For definitions of PPE size (PE-, PE+, PE+1, PE+2 and P+3), see text.
