# Supplementary material for: Population-Based Cohort of Children With Parapneumonic Effusion and Empyema Managed With Low Rates of Pleural Drainage
Source: Front Pediatr. 2021 Jul 21;9:621943. doi: 10.3389/fped.2021.621943 (PMC8335639; doi:10.3389/fped.2021.621943)
Supplement: Supplementary file 5 [file Table_5.DOCX]

**Table S5**. Triennial trend in length of intravenous treatment, fever and hospital stay in children hospitalized with parapneumonic pleural effusion (PPE)

| YEARs | 2010-2012 | 2013-2015 | 2016-2018 | p-value |  |
| --- | --- | --- | --- | --- | --- |
| Length of intravenous treatment | | | | | |
| Total PPE (n = 307) | 7 (4-11) | 7 (4-12) | 7.5 (4-12) | 0.892 |  |
| Total PE+ (n = 157) | 11 (7-14) | 12 (8-14.5) | 11 (8-14) | 0.510 |  |
| - PE+1 (n= 66) | 10 (6-11) | 8 (5-11.5) | 10 (7-11.5) | 0.897 |  |
| - PE+2 (n = 55) | 12 (8-14) | 12 (9-14) | 11 (10-12.5) | 0.898 |  |
| - PE+1 & PE+2 (n = 121) | 10.5 (7-13) | 11 (7-13) | 10 (7-12) | 0.962 |  |
| - PE+3 (n = 36) | 15 (9-16) | 14.5 (13-16) | 13 (12-15.5) | 0.692 |  |
| Length of fever | | | | | |
| Total PPE (n = 302) | 3 (1-6) | 4 (2-7) | 3 (1-7) | 0.348 |  |
| Total PE+ (n = 152) | 6 (2-9.5) | 7.5 (5-10) | 6 (3-10.5) | 0.162 |  |
| - PE+1 (n = 63) | 5 (2-7) | 5 (3-8) | 3 (1-5) | 0.430 |  |
| - PE+2 (n = 55) | 7 (3-11) | 7 (5-10.5) | 8 (5-10.5) | 0.922 |  |
| - PE+1 & PE+2 (n = 118) | 6 (2-9.5) | 6 (4-10) | 5 (2-8) | 0.397 |  |
| - PE+3 (n = 34) | 5.5 (1-10.5) | 10 (8.5-13) | 11 (8.5-17) | 0.067 |  |
| Length of hospital stay | | | | | |
| Total PPE (n = 318) | 7 (5-12) | 7 (4-13) | 8 (5-12) | 0.914 |  |
| Total PE+ (n = 161) | 10 (7-14.5) | 12 (8-15) | 11 (8-15) | 0.430 |  |
| - PE+1 (n = 68) | 9 (6.5-13) | 10 (6-14) | 9 (7-12) | 0.990 |  |
| - PE+2 (n = 57) | 12 (8-15) | 11.5 (9-15.5) | 12 (10.5-14) | 0.903 |  |
| - PE+1 & PE+2 (n = 125) | 10 (7-13) | 11 (8-14) | 11 (7-14) | 0.814 |  |
| - PE+3 (n = 36) | 17 (10-26) | 15 (13.5-17.5) | 15 (11-21) | 0.984 |  |

The cells express the median (and interquartile range) in days in each 3-year period and each group.

For definitions of PPE size (PE-, PE+, PE+1, PE+2 and P+3), see text.
