# Supplementary material for: Population-Based Cohort of Children With Parapneumonic Effusion and Empyema Managed With Low Rates of Pleural Drainage
Source: Front Pediatr. 2021 Jul 21;9:621943. doi: 10.3389/fped.2021.621943 (PMC8335639; doi:10.3389/fped.2021.621943)
Supplement: Supplementary file 6 [file Table_6.DOCX]

**Table S6**. Frequency of baseline and treatment explanatory variables, and mean length of hospital stay in patients with parapneumonic pleural effusion ≥ 10 mm (PE+)

|  | | Total | | Length of hospital stay | | |
| --- | --- | --- | --- | --- | --- | --- |
|  | | n | % | Mean | SD | p-value |
| **BASELINE VARIABLES** | | | | | | |
| Sex | Female | 74 | 46.0% | 12.9 | 7.9 | 0.837 |
|  | Male | 87 | 54.0% | 12.0 | 6.3 |  |
| Year of admission | 2010-2012 | 59 | 36.6% | 11.6 | 5.9 | 0.430 |
|  | 2013-2015 | 49 | 30.4% | 12.9 | 7.7 |  |
|  | 2016-2018 | 53 | 32.9% | 12.9 | 7.6 |  |
| Trimester of admission | T3 | 16 | 9.9% | 9.4 | 4.3 | 0.014* |
|  | T4 | 46 | 28.6% | 10.8 | 5.7 |  |
|  | T1 | 63 | 39.1% | 13.3 | 6.3 |  |
|  | T2 | 36 | 22.4% | 14.3 | 9.8 |  |
| First hospital admission | RH | 35 | 21.7% | 12.4 | 8.0 | 0.381 |
|  | CH1 | 24 | 14.9% | 14.7 | 12.4 |  |
|  | CH2 | 21 | 13.0% | 11.3 | 3.5 |  |
|  | CH3 | 18 | 11.2% | 10.1 | 5.4 |  |
|  | CH4 | 17 | 10.6% | 12.4 | 3.8 |  |
|  | CH5-8 | 46 | 28.6% | 12.7 | 4.8 |  |
| Size of effusion | PE+1 | 68 | 42.2% | 9.4 | 4.4 | <0.001* |
|  | PE+2 | 57 | 35.4% | 12.4 | 5.0 |  |
|  | PE+3 | 36 | 22.4% | 18.1 | 10.0 |  |
| Complicated effusion | Yes | 113 | 70.2% | 14.1 | 7.4 | <0.001* |
|  | No | 30 | 18.6% | 9.1 | 3.9 |  |
|  | Unknown | 18 | 11.2% | 7.6 | 4.5 |  |
| Neurological diseases | No | 152 | 94.4% | 12.6 | 7.2 | 0.193 |
|  | Yes | 9 | 5.6% | 9.3 | 4.2 |  |
| Asthma | No | 145 | 90.1% | 12.7 | 7.2 | 0.147 |
|  | Yes | 16 | 9.9% | 9.8 | 4.8 |  |
| Antibiotics before | No | 83 | 52.5% | 13.6 | 6.7 | 0.001* |
| admission | Yes | 75 | 47.5% | 10.8 | 7.0 |  |
| Confirmed positive | No | 142 | 88.2% | 11.6 | 5.9 | 0.001* |
| culture | Yes | 19 | 11.8% | 18.7 | 11.1 |  |
| Affected lung | Left | 83 | 51.6% | 12.1 | 5.1 | 0.541 |
|  | Right | 78 | 48.4% | 12.8 | 8.7 |  |
| Need for oxygen | No | 76 | 48.7% | 10.2 | 4.9 | <0.001* |
|  | Yes | 80 | 51.3% | 14.5 | 8.3 |  |
| **TREATMENT VARIABLES** | | | | | | |
| Transfer to RH^a^ | No | 50 | 40.7% | 9.8 | 4.4 | <0.001* |
|  | Yes | 73 | 59.3% | 14.2 | 7.7 |  |
| Chest tube pleural | No | 125 | 77.6% | 10.6 | 4.5 | <0.001* |
| drainage | Yes | 36 | 22.4% | 18.7 | 10.2 |  |
| Intensive care | No | 147 | 91.3% | 11.8 | 6.1 | 0.002* |
|  | Yes | 14 | 8.7% | 19.1 | 11.8 |  |
| Mechanical ventilation | No | 155 | 96.3% | 12.0 | 6.2 | 0.007* |
|  | Yes | 6 | 3.7% | 24.0 | 15.8 |  |

T3: July-September; T4: October-December; T1: January-March; T2: April-June. RH: reference hospital. CH: community hospital

For definitions of parapneumonic effusion size (PE+, PE+1, PE+2 and P+3), see text.

^a^ Only including 123 patients firstly admitted to a CH.

* p < 0.05
