# Supplementary material for: Population-Based Cohort of Children With Parapneumonic Effusion and Empyema Managed With Low Rates of Pleural Drainage
Source: Front Pediatr. 2021 Jul 21;9:621943. doi: 10.3389/fped.2021.621943 (PMC8335639; doi:10.3389/fped.2021.621943)
Supplement: Supplementary file 7 [file Table_7.DOCX]

**Table S7**. Descriptive baseline explanatory variables and correlation to the length of hospital stay in patients with parapneumonic pleural effusion ≥ 10 mm (PE+)

|  | n | Minimum | Maximum | Mean | SD | Spearman’s ρ^1^ | p-value |
| --- | --- | --- | --- | --- | --- | --- | --- |
| Length of hospital stay (days) | 161 | 1.0 | 54.0 | 12.4 | 7.1 | - | - |
| Age (years) | 161 | 0.0 | 14.0 | 4.4 | 3.4 | -0.233 | 0.003* |
| Fever before admission (days) | 156 | 0.0 | 14.0 | 4.0 | 2.7 | 0.080 | 0.321 |
| Antibiotics before admission (days) | 154 | 0.0 | 11.0 | 1.9 | 2.7 | -0.310 | <0.001* |
| Leucocytes (10^9^/L) | 158 | 2.0 | 48.0 | 21.1 | 9.4 | 0.273 | 0.001* |
| Neutrophils (10^9^/L) | 156 | 0.6 | 39.7 | 15.9 | 8.4 | 0.242 | 0.002* |
| C-reactive protein (mg/dL) | 158 | 0.1 | 70.7 | 24.2 | 13.8 | 0.400 | <0.001* |
| Sodium (mmol/L) | 147 | 123.0 | 145.0 | 134.0 | 3.7 | -0.254 | 0.002* |
| Urea (mg/dL) | 144 | 2.4 | 88.0 | 28.2 | 15.7 | 0.208 | 0.012* |

^1^ Spearman's rank correlation coefficient to the length of hospital stay; * p < 0.05
