# Supplementary figures and images for: Population-Based Cohort of Children With Parapneumonic Effusion and Empyema Managed With Low Rates of Pleural Drainage
Source: Front Pediatr. 2021 Jul 21;9:621943. doi: 10.3389/fped.2021.621943 (PMC8335639; doi:10.3389/fped.2021.621943)

**Figure S1**.Goodness of fit of multivariate Gamma model and influence variables


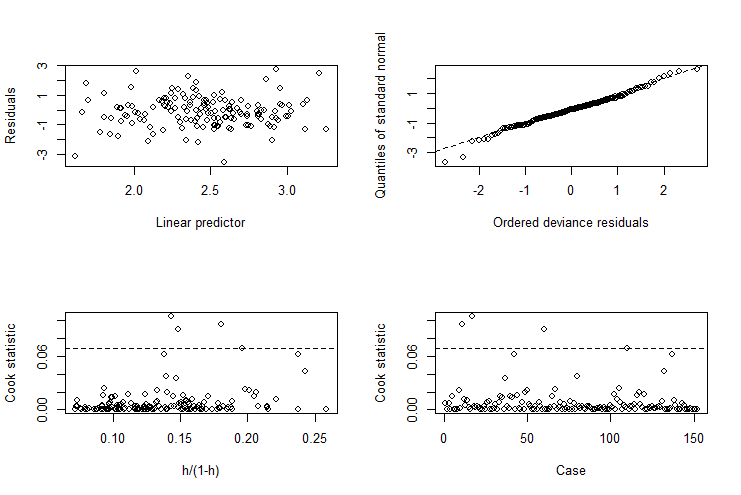

Supplement: Supplementary file 8 [file Data_Sheet_1.DOCX]
